# No excuses - improving child public health outcomes in humanitarian settings requires reliable data

**DOI:** 10.1371/journal.pgph.0004231

**Published:** 2025-03-14

**Authors:** Ayesha Kadir, Amy J. Stevens, Paul H. Wise, Rudzani Muloiwa

**Affiliations:** 1 Viborg Regional Hospital, Viborg, Denmark; 2 Yorkshire and Humber School of Public Health, Leeds, United Kingdom; 3 Stanford University School of Medicine, Stanford, California, United States of America; 4 Department of Paediatrics & Child Health, Red Cross War Memorial Children’s Hospital, University of Cape Town, Cape Town, South Africa; PLOS: Public Library of Science, UNITED STATES OF AMERICA

Advances in medical and public health knowledge and technology have supported extraordinary improvements in humankind’s ability to understand, diagnose, treat, and cure diseases and health conditions. These advances have been dependent upon well-constructed public health interventions, which have in turn relied on data to inform priorities, intervention design, monitoring, and evaluation. This paper argues that public health interventions for children in humanitarian settings can and should be made using sound evidence. Beginning with a brief overview of child public health (Box 1) [1], we review the main challenges to collecting child public health data in humanitarian settings. We argue that actors delivering interventions for children and actors involved in data collection hold a moral responsibility to measure the impacts of their activities on children, and we provide some suggestions for the way forward based on a recent scoping review of child public health indicators in fragile, conflict-affected, and vulnerable (FCV) settings [2].

## Child public health – a useful approach to improve interventions for children in humanitarian settings

By its nature, child public health includes a range of disciplines focused on, related to, or impacting children’s and young people’s health. The importance of including the perspectives, views, and priorities of children and young people is considered an essential component of child public health work. It is not only the right thing to do, but also necessary to meaningfully understand and effectively respond to their needs. Child public health provides a perspective that can allow us to see children in the context of their lives and consider what this means for their health status, risks, and needs both in the present and as they grow.

**Box 1. Child public health**

**Child public health** includes a range of disciplines that focus on the study of the patterns of health and illness in children and young people, factors which affect their health, and interventions by individuals, groups, organizations, and societies to modify health determinants and improve the health and wellbeing of young people. A hallmark of child public health is that it is rooted in child rights and holds at its core the ideal of achieving the optimal health and wellbeing of all young people, regardless of their identities [1].

The field of child public health has gained traction in the past few decades, as seen by a significant and growing body of evidence for the determinants of children’s health and the impacts of adversity on a child’s health and development across their life course. However, child public health is still not well understood by many public health professionals, donor agencies, or humanitarian workers in crisis contexts. This is reflected in a siloed approach to humanitarian interventions targeting children [3], and the resulting limitations in our ability to promote, protect, and respond to the health of children in humanitarian contexts [4]. For example, after treatment for severe acute malnutrition (SAM), children are often discharged to conditions that place them at high risk for relapse. Across settings, children discharged after treatment for SAM carry heightened morbidity and mortality risk in the following 24 months [5]. It is not common practice for health workers in humanitarian settings to ascertain whether a newly admitted adult patient is a caregiver of children or to ensure that someone suitable has taken responsibility to meet those children’s needs, including safety [6]. Recommendations for paediatric services in humanitarian health responses provide limited guidance on how to ensure treatment spaces for children are safe and child-friendly, such as provision of adequate supervision of children admitted without a caregiver, ensuring toddlers and young children do not have access to medicines, instruments, or chemicals that could harm them, or creation of spaces in the facility for children to play and learn once they are clinically well enough to do so [7]. The latter two examples are particularly important during outbreak responses, where children may be separated from caregivers during admission (of the child and/or caregiver) to an isolation and treatment centre. There are recent examples of guidance to address some of these gaps, such as the brief on Nurturing Care for children living in humanitarian settings [8], and a series of tools from the READY Initiative to support child protection during infectious disease outbreaks [9]. However, implementation of these tools is not yet routine. Recent efforts to incorporate children’s priorities into needs assessments and to strengthen accountability mechanisms to children in humanitarian interventions are also very encouraging, but there is some way to go before it becomes standard practice [10].

Humanitarian responses often include interventions from a range of sectors that effectively support child public health. These sectors include health; nutrition; mental health and psychosocial support; water, sanitation & hygiene; education; (child) protection; social and behaviour change; shelter; food security and livelihoods; and cash and voucher assistance [11]. Non-health sectors [12] play important roles in recognising and responding to structural and social determinants of child health. Examples include provision of resources to meet children’s and families’ basic needs, reuniting separated and orphaned children with family, improving safety, creating and restoring learning environments, striving to limit interpersonal violence exposure, and supporting referral pathways to link children and families with relevant services [3]. Robust baseline child public health data would improve our ability to design and deliver relevant, appropriate, just, and effective responses when a crisis occurs. Coordinated child public health data practices between governments, aid agencies, and humanitarian responders, would improve our ability to ascertain the outcome of interventions on children’s health and development throughout a crisis and during the recovery period.

Child public health can be useful for humanitarian workers. A child public health approach to humanitarian response could support meaningful integration of interventions across sectors, promote evidence-based action, support the measurement of outcomes, and foster accountability to children.

## An urgent need for reliable data to inform practice

Delivering on child public health is highly dependent on the availability of high-quality data, including data measured over extended periods of time. The improved capture and analysis of data have changed how human health is viewed. Data from longitudinal studies have scientifically demonstrated what was implicitly understood by health workers for centuries about the profound and complex influence of social and structural factors on health outcomes [13,14]. Data have helped improve understanding on how responses to social and structural determinants can be best formulated to improve health outcomes – and especially the need to invest in the early years [13].

It is encouraging to see new methods developed to better understand the risks, needs, and priorities of hard-to-reach populations in places affected by crises, such as mapping conflict intensity to Demographic and Health Survey data [15]. However, while clear patterns in health risks and needs have emerged, conspicuous gaps in our knowledge persist. The gaps indicate a need for reflection on what information we prioritise and how we make decisions about which data are collected and how they are utilised. Humanitarian organisations working in public health often focus on collecting process and output indicators, which are easier to collect, rather than outcomes [16]. Further, public health data tend to focus on specific populations while ignoring others or lumping them into a larger whole. This is notably the case for routine data on children and young people in humanitarian settings, despite a longstanding and well-evidenced knowledge about the marked diversity of health risks and needs among children and young people according to their age, stage of development, living environment, and exposure to trauma and adversity.

Before reviewing some of the challenges for child public health data collection in humanitarian settings, it is important to acknowledge broader challenges and barriers to high quality and comparable child health data that are also found in stable settings and the ongoing activities to address these. Among the latter are three World Health Organization Technical Advisory Groups (TAGs) working to improve the measurement of maternal and newborn, child and adolescent health, and support the coordination of initiatives to improve the measurement of outcomes. The work of these TAGs has supported a considered call for standardised age disaggregation [17].

The legal definition of child varies by country, while the Convention on the Rights of the Child defers to domestic law on who constitutes a child [18]. Age disaggregation is therefore the first step to making children and young people visible in data. Standardised age disaggregation would improve the assessment of age-specific health patterns for children and young people. If standard child public health indicators were measured by different actors, pooling and comparison of data across settings would be possible. Pooling child public health data could allow us to better understand context-specific patterns as well as global patterns of child health and burden of disease in children. In current practice, age disaggregation is not yet standardised, and child health indicators, even if reliably measured in different settings, are not routinely shared between actors [2].

While the work to improve data quality and standardise child health data gains momentum, children in humanitarian settings are being left behind. This gap is notable because children account for a large proportion of the population in most crisis-affected areas, and they are disproportionately likely to be affected by crises. Children account for 40% of forcibly displaced people, but only 30% of the global population [19]. One in six children live within 50 km of an active conflict zone [20]. One billion children – nearly half of all children globally – are estimated to be at extremely high risk to suffer harmful consequences from climate change [21]. The low visibility of children in public health data in crisis-affected contexts means that our understanding of their health risks and needs is largely based on limited routine data, intermittent surveys, and experience; these serve as a weak foundation for decision-making for humanitarian response. It is therefore unsurprising that humanitarian health interventions for children are not fit for purpose [4].

## Moral accountability to document children’s health and mortality

Working with data - in any way and at any level - is a moral act, and its collection and analysis, a moral imperative [22]. At the most basic level, data help us to recognise the existence or absence of something or someone. Data also help us to understand characteristics, associations, and trends. In more sophisticated experiments, we can discern cause and effect. Qualitative data bring depth and nuance to our understanding by capturing information on history, context, perceptions, beliefs, behaviours, and more. However, at the end of the day, what has been measured and our understanding of it, largely reflects the questions that were asked, the kinds of information gathered, and the way we have made sense of it all. Knowledge is ultimately the product of assumptions, biases, values, and priorities that are brought into the entire process. We even have the potential to look away when we choose. With the rapid expansion of the peer-reviewed literature, conspicuous gaps suggest that we do sometimes look away [23,24]. For example, there is a notable geographic concentration of evidence on the impacts of armed conflict on child health – indicating that some children’s lives are considered more valuable than others [23].

Mortality is the most basic measurement of child public health, yet even this remains a challenge in humanitarian settings [25,26]. We argue that the challenges in measuring child deaths are, in most contexts, less an issue of feasibility, and more of a collective choice through failure to collaborate across agencies and sectors using the existing tools and resources. The recent scoping review of child public health indicators in FCV settings identified 152 routinely collected situation, impact, and outcome indicators, of which 29 were mortality indicators [2]. The scoping review findings indicate that measurement of children’s deaths is possible in even some of the most challenging settings [2]. Yet there are no actual figures for the number of children dying across settings for any reason – only estimates.

Counting the dead, ill, and injured children is the first step in recognising our moral accountability for the harms that children suffer in humanitarian settings, whether these are directly caused by the main crisis or crises, or indirectly through downstream effects [25]. Understanding the scale of what has happened and what has been done is necessary to make sound decisions about how to respond. The counting of the dead will be done by health workers; adequate support and coordination for sharing and pooling of data is necessary to make this possible. Governments and other institutions that hold power and influence are increasingly using data to set priorities, justify actions, and allocate funding. In this time of increasing demand for evidence to inform decisions, justice in data practices is more important than ever. Justice includes ensuring the visibility of children and marginalised groups in data, and ensuring their participation in setting measurement priorities [27,28]. It is an issue of respecting the lives, including the extinguished lives, of all children.

## Measuring child public health outcomes in humanitarian settings

In humanitarian contexts, routine public health outcome data collection is hampered by a range of factors. Challenges include the destruction of health and public health infrastructure; population movements, including displacement of health workers; limited or interrupted access to affected populations; short-term funding for humanitarian interventions; siloed working; and competition between sectors and operational agencies for limited resources [16,29–31]. To overcome some of these challenges and improve the effectiveness and reach of interventions, integration of responses by different sectors and coordination of interventions in the same geographic location are becoming increasingly common [32,33]. Multi-cluster interagency rapid needs assessments at the onset of a crisis have become standard practice [34]. Efforts are underway to develop adaptations of outcome indicators for contexts where the gold standard of measurement is not feasible [16]. This work is important, not least because it recognises that it is unacceptable to neglect measuring outcomes solely because the most rigorous methods are not practically feasible in humanitarian settings.

While the move towards integration of needs assessments and interventions is a step in the right direction, data practices remain largely siloed. As a result, existing data struggle to capture the health impacts of social determinants of child health and the protective effects of non-health interventions on children’s health outcomes. In the health sector, a framework has been developed to support monitoring and evaluation for sexual, reproductive, maternal, newborn, child, and adolescent health services and outcomes in humanitarian settings [35]. Similarly, interagency groups have developed recommended indicators for actors working in specific sectors, many of which are child public health indicators. Some notable examples have been developed by groups working in child protection [36] and education [37]. In addition, each cluster has developed recommended indicators, many of which are relevant to children and young people [38]. When we review these indicators with a child public health lens, however, the limitations in the current approach begin to take form. Hundreds of indicators exist, many of which are similar, but not the same. Additionally, there important gaps in measured indicators. The scoping review by Kadir et al found that there is not a single outcome or impact indicator on early child development that is routinely collected by 5 large operational agencies, required by major donors, or recommended by technical groups and partnerships [2]. Integration also remains challenging - there are few education-related indicators used outside of the education sector, and few child protection indicators used outside of the child protection sector. Yet, the evidence for the short- and long-term harms to health and development from severe adversity in childhood are well-described [39]. Alterations in child development have implications not only for children’s health and disability, but also their access to education, and a host of protection risks. These factors are not isolated; they intersect to influence a child’s medium and long-term health, development, and life trajectory. The rapid development of young children also means that we have an opportunity to track development with short intervals, especially during the first few years of life, and to monitor the impacts of both health and non-health interventions on child development outcomes in the short term.

## The way forward: fewer and better indicators

The large number of impact and outcome indicators that are routinely collected by aid agencies is proof that it is possible to routinely collect these data. Challenges in data collection in humanitarian settings are therefore not a valid excuse to justify not collecting child public health outcome data. However, more data is not necessarily better. With limited resources and numerous actors working across a range of sectors, it is more appropriate to identify a short list of indicators and develop process to support pooling and sharing of data.

A formal, intersectoral process is needed to develop a of short list of key high-quality, integrated child public health outcome indicators for routine measurement in humanitarian settings. Children and young people should be included in this process alongside governmental, United Nations, and nongovernmental actors working in sectors that support child public health. We suggest that this process begin with the identification and prioritisation of critical information needs for the purpose of operational decision-making across the different phases of crisis response, from prevention to preparedness, response and recovery. Subsequent identification and/or development of a shortlist of indicators should be based on the prioritised operational needs. Proxy indicators should be developed for periods when the gold standard indicators are not feasible to collect.

Taking a child public health approach to humanitarian response could markedly improve our understanding of children and young people’s health needs, and interventions to support and protect child health in a given context. As such, child public health could improve the impact of humanitarian responses to support children, and ultimately, child health outcomes in the immediate- and long-term. Indeed, taking a child public health approach would likely change what we prioritise and what we do to support children and young people living in crisis contexts.
